# KIAA0101 Is Overexpressed, and Promotes Growth and Invasion in Adrenal Cancer

**DOI:** 10.1371/journal.pone.0026866

**Published:** 2011-11-11

**Authors:** Meenu Jain, Lisa Zhang, Erin E. Patterson, Electron Kebebew

**Affiliations:** Endocrine Oncology Section, Surgery Branch, National Cancer Institute, Bethesda, Maryland, United States of America; Enzo Life Sciences, Inc., United States of America

## Abstract

**Background:**

KIAA0101 is a proliferating cell nuclear antigen-associated factor that is overexpressed in some human malignancies. Adrenocortical neoplasm is one of the most common human neoplasms for which the molecular causes are poorly understood. Moreover, it is difficult to distinguish between localized benign and malignant adrenocortical tumors. For these reasons, we studied the expression, function and possible mechanism of dysregulation of KIAA0101 in human adrenocortical neoplasm.

**Methodology/Principal Findings:**

KIAA0101 mRNA and protein expression levels were determined in 112 adrenocortical tissue samples (21 normal adrenal cortex, 80 benign adrenocortical tumors, and 11 adrenocortical carcinoma (ACC). SiRNA knockdown was used to determine the functional role of KIAA0101 on cell proliferation, cell cycle, apoptosis, soft agar anchorage independent growth and invasion in the ACC cell line, NCI-H295R. In addition, we explored the mechanism of KIAA0101 dysregulation by examining the mutational status. KIAA0101 mRNA (9.7 fold) and protein expression were significantly higher in ACC (p<0.0001). KIAA0101 had sparse protein expression in only a few normal adrenal cortex samples, which was confined to adrenocortical progenitor cells. KIAA0101 expression levels were 84% accurate for distinguishing between ACC and normal and benign adrenocortical tumor samples. Knockdown of KIAA0101 gene expression significantly decreased anchorage independent growth by 80% and invasion by 60% (p = 0.001; p = 0.006). We found no mutations in KIAA0101 in ACC.

**Conclusions/Significance:**

KIAA0101 is overexpressed in ACC. Our data supports that KIAA0101 is a marker of cellular proliferation, promotes growth and invasion, and is a good diagnostic marker for distinguishing benign from malignant adrenocortical neoplasm.

## Introduction

Adrenal neoplasms are one of the most common human neoplasms, often detected incidentally [1]. Most adrenal neoplasms are benign but it is often difficult to exclude a malignant tumor such as adrenocortical carcinoma. Adrenocortical carcinoma (ACC) is a rare malignancy of the adrenal cortex with a poorly understood mechanism of development, and a dismal patient outcome due to a lack of effective therapy [2]. The annual incidence of ACC is approximately 1–2 cases per million [3], [4], [5]. Complete surgical resection is the only possible curative therapy but over two-thirds of patients present with metastatic disease and even those patients who have complete resection develop recurrent disease in over 50% of cases [2], [6]. Patients with metastatic disease have a five-year survival rate of less than 10% and those with recurrent disease have a five-year survival of only 50% [2], [6].

ACC may be associated with hereditary cancer syndromes such as Beckwith-Wiedemann syndrome (associated with germline 11p15 chromosomal alterations leading to *IGF2* overexpression, OMIM #130650), Li-Fraumeni syndrome (*TP53* mutation, OMIM #151623), multiple endocrine neoplasia type 1 (mutations in the menin tumor suppressor gene, OMIM #131100), and Gardner's syndrome (*APC* mutation, OMIM #175100). The genetic changes associated with hereditary cancer syndromes have provided important information about the possible molecular mechanisms involved in ACC. However, most cases of ACC are sporadic, and the molecular events that lead to initiation and progression of adrenocortical tumors remain unclear.

Genome–wide gene expression profiling provides important insight into the molecular pathways that are dysregulated in cancer and may be involved in tumor initiation and progression. Since the molecular mechanism of adrenocortical carcinogenesis is poorly understood, cDNA microarray analysis of adrenocortical tumors has been used to reveal genes whose misexpression is associated with ACC [7], [8], [9], [10], [11]. Among the genes that were found to be upregulated in ACC, KIAA0101 (also known as L5; PAF; OEATC1; NS5ATP9; OEATC-1; p15) is a novel potential diagnostic and prognostic marker, and target for ACC therapy. KIAA0101 encodes a proliferating cell nuclear antigen (PCNA) -associated factor with an unknown function. KIAA0101 is overexpressed in human malignancies such as hepatocellular and pancreatic carcinoma [12], [13], [14], [15], [16], [17], [18], [19], [20], [21], [22]. However, the role of KIAA0101 in cancer and the mechanism leading to dysregulated expression of KIAA0101 is unclear.

In this study, we addressed these issues and showed that KIAA0101 is overexpressed in ACC and a marker of cellular proliferation. Furthermore, reducing KIAA0101 expression in ACC resulted in growth suppression and invasion suggesting that KIAA0101 plays an oncogenic role in ACC.

## Methods

### Tissue specimens

The National Cancer Institute review board approved this research protocol after informed written consent was obtained from all participants. Adrenal tissues were snap frozen at the time of surgery and stored at −80°C. In this study, 112 human adrenocortical tissue specimens were analyzed including 21 normal adrenocortical tissues, 80 benign adrenocortical tumors, and 11 primary adrenocortical carcinomas(78 of the benign adrenocortical tumors and 11 of the primary adrenocortical carcinomas were previously analyzed) [11]. An independent set of ACC metastases (n = 29) and recurrences (n = 2) was also used for validation. The diagnosis of unequivocal ACC and benign adrenocortical tumor was confirmed in all cases by histologic examination and clinical follow up. The average follow-up time was 26.3 months for patients with benign adrenocortical tumors and 44.4 months for patients with ACC.

### Cell culture

The NCI-H295R ACC cell line (ATCC, Rockville, MD) was grown and maintained in DMEM supplemented with 1% ITS+ Premix (BD Biosciences, San Jose, CA), 2.5% Nu-Serum I (BD Biosciences), and 10,000 U/mL penicillin/streptomycin in a standard humidified incubator at 37°C in a 5% CO_2_ atmosphere. Twenty-four hours after cells were seeded in 24 and 6 well plates (4×10^^4^ cells in 0.5 ml and 1.6×10^^5^ cells in 2 ml), cells were transfected with a nonspecific negative control siRNA and with a KIAA0101 specific siRNA at a final concentration of 40 and 80 nM (AM4636 and AM46235, respectively, Applied Biosystems, Foster City, CA). The TransIT siQuest reagent (Mirus Bio, LLC) was used to deliver siRNA to the cells according to manufacturer instructions. The knockdown efficiency was similar for 40 and 80 nM. All in vitro assays were done with both 40 and 80 nM concentration of KIAA0101 specific siRNAs and nonspecific negative control.

### RNA and Protein Preparation

RNA was isolated using the TRIzol reagent following the manufacturer's instructions (Invitrogen Inc., Carlsbad, CA). RNA quantity and quality was assessed by using NanoDrop (NanoDrop Technologies, Inc., Thermo Fischer) and Agilent 2100 Bioanalyzer (Agilent Technologies, Santa Clara, CA), respectively.

RIPA buffer was used to prepare tissue lysates and whole cell lysate was prepared with 1% SDS plus 10 mM Tris [pH 7.5] buffer. The protein concentration was determined using the BioRad RC DC protein assay (Hercules, CA).

### Real time quantitative reverse-transcription polymerase chain reaction (RT-PCR)

Total RNA (125 ng) was reverse transcribed using the RT script cDNA synthesis kit (USB Corporation, Cleveland, OH). Real time quantitative PCR was used to measure mRNA expression levels relative to glyceraldehyde-3-phosphate dehydrogenase (GAPDH) mRNA expression. Normalized gene expression level  =  2 ^– (C^t^ of gene of interest – C^t^ of GAPDH)^×100%, where C_t_ is the PCR cycle threshold. The PCR primers and probes for KIAA0101 (Hs00207134_m1) and GAPDH (Hs99999905_m1) were obtained from Applied Biosystems (Assay-on-Demand kit®, Foster City, CA). All PCR reactions were performed in a final volume of 20 µl with 1 µl of cDNA template on an ABI PRISM®7900 Sequence Detection System (Applied Biosystems). The PCR thermal cycler condition was 95°C for 12 minutes followed by 40 cycles of 95°C for 15 seconds and 60°C for 1 minute. All experiments were performed in triplicate and repeated three times.

### Western blot analysis

Protein samples (40 µg) were separated in 4% to 20% SDS-PAGE gel and transferred onto a nitrocellulose membrane (Amersham Pharmacia Biotech, Piscataway, NJ). Western blotting was performed following standard procedures using primary mouse monoclonal antibodies, anti-KIAA0101 (Abcam; ab56773) at 1∶100 dilution and anti-β-actin (sc-81178, Santa Cruz Biotechnology Inc, Santa Cruz, CA) at 1∶2,000 dilution. Signal detection was performed using HRP conjugated secondary antibody and an enhanced chemiluminescence kit (Amersham Pharmacia Biotech).

### Immunohistochemical (IHC) and Immuunofluoroscence staining

Tumor tissues were formalin fixed, embedded in paraffin, and 5 micron thick sections were cut for immunostaining. Sections were incubated with the primary anti-KIAA0101 mouse monoclonal antibody at 1∶300 dilution overnight at 4°C (Abcam; ab56773) followed by biotinylated secondary antibody for 1 hr at room temperature (1∶150; Vector Laboratories, Burlingame, CA, USA). Sections were developed using 3,3′-diaminobenzidine DAB as the chromogen (ABC elite kit, Vector Laboratories, Burlingame, CA, USA) and hematoxylin as counterstain. The sections were rehydrated and mounted with vectamount mounting medium (Vector Laboratories, Burlingame, CA, USA). The slides were scanned under Olympus light microscope (Nikon, Tokyo, Japan) and pictures were taken at 20X and 40X magnifications. A semiquantitative scoring system was used to analyze KIAA0101 expression; expression level was classified as no staining (0), <30% of cells staining (1), 30–50% cells (2) and >50% of cells. Two observers, who were blinded to the tumor type, independently scored each sample. The scores were averaged to obtain the final KIAA0101 expression score.

Immuunofluoroscence staining was done using primary anti-KIAA0101mouse monoclonal antibody at 1∶300 dilution overnight (Abcam; ab 56773) and rabbit anti-SF-1 (Millipore; 07-618, 2 µg/ml) at 4°C. The primary antibodies were detected with fluorophore conjugated with RedTX anti-rabbit IgG (Invitrogen, Carlsbad, CA) and FITC anti-mouse IgG secondary antibodies (Vector Laboratories). The slides were then rinsed and mounted with DAPI (4′,6-diamidino-2-phenylindole) mounting solution. Images were analyzed with a Zeiss Axioskop-2 microscope at 20X and 40X magnifications.

### DNA Purification

Genomic DNA was isolated from 1 mg of tumor and normal samples using the QIAamp DNA Blood Mini Kit (Qiagen, Hilden, Germany), eluted in a total volume of 200 µL and stored at −20°C. DNA concentrations were measured by UV absorbance using NanoDrop (NanoDrop Technologies, Inc., Thermo Fischer).

### Sequencing KIAA0101 coding region

The KIAA0101 exons 1, 2, 3 and 4 were sequenced. PCR primers were designed for coding region using UCSC genome browser, Primer 3 integrated software. The primer sequences are listed in **Table 1**. Primers were made by IDT, Inc. (Coralville, IA). Exons 1, 2, 3 and 4 were amplified as three separate reactions in 50 µl using PCR master mix (Fermentas Inc. USA). The standard PCR conditions were initial denaturation at 95°C for 5 min followed by 30 cycles of denaturation at 95°C for 5 min, annealing at 50°C for 1 min, extension at 72°C for 1 min and final extension at 72°C for 10 min. PCR products were visualized on 2% agarose gel. The products were purified using standard Exo-nuclease I and FastAP (Fermentas Inc.). Purified PCR products were sequenced using ABI sequencer 3730xl with big dye method (DNA Sequencing kit, big dye terminator cycle sequencing; Applied Biosystems). Mutations were analyzed by the Mutation Surveyor V 3.3 Programme (Soft Genetics; www.softgenetics.com). All of the sequencing data has been deposited in GenBank.

**Table 1:** Genomic PCR Primer sequences for KIAA0101 exons.

Genomic Primer Sequences	PCR product size in bp
**Exon 1–2**	
F CCAATATAAACTGTGGCGGG	617
R AAATTCGGGCGTGAGTACC	
**Exon 3**	
F CCTTTGAGAATTTGATGTTAAAGAAG	358
R TGGCCTCAAGTGATCCTC	
**Exon 4**	
F ACAACGTAGTCTAAAGGAGAAACACTG	412
R AATTAAATGCCTGTTCAACAAAG	

### Cell proliferation

Cells were seeded in a 96-well plate at a concentration of 5×10^^3^ cells per 100 µL culture medium in six replicates. At each timepoint, the media was aspirated from the well and the cells were immediately frozen at −80°C for 24 hours. The plates were thawed at room temperature, and prepared for cell number quantification using the CyQUANT™ assay kit (Invitrogen, Carlsbad, CA). The CyQuant assay was performed according to the manufacturer's instructions, and analyzed on a fluorometric microplate reader (Molecular Devices, Sunnyvale, CA) at 480 nm/520 nm.

### Flow cytometry and apoptosis analyses

KIAA0101 and negative control siRNA treated cells were harvested, ethanol-fixed overnight at 4°C, and resuspended in 1x PBS to a concentration of 1×10^^6^ cells/mL. Cells were treated with DNase-free RNase (100 µg/ml) for 20 min at 37°C. The cells were stained with propidium iodide at concentration of 50 µg/ml and samples were stored at 4°C. Flow cytometric analysis was performed on a Becton Dickinson FACScan (BD Biosciences, Franklin Lakes, NJ). Data files were generated for 20,000 events (cells) using the CellQuest software. The fraction of the total cell population present in each of the G1, S and G2/M cell cycle phases was obtained from ModFit LT software (Verity Software House, Inc.). Apoptosis analysis was performed using Annexin V staining (ApoAlert® Annexin V Apoptosis Kit) according to the manufacturer's instruction (Clontech, Mountain View, CA).

### Soft agar anchorage independent growth assay

Two-layered soft agar assays were performed in six-well plates. The bottom layer of agar (2 ml/well) contained 0.5% agar (Difco Noble Agar, Becton, Dickinson and Company, Sparks, MD) in maintenance medium. Five days after siRNA transfection, cells were trypsinized, counted, and 50,000 cells were mixed with 1.3 ml of top agar solution supplemented with 10% Nu-serum (0.3% agar in culture media). Solidified agar was overlayed with 1 ml of culture media containing 10% Nu-serum. The plates were cultured at 37°C in 5% CO_2,_ and the media was changed twice a week. After 16 days of culture, cell colonies were stained with 0.2% crystal violet solution and examined by microscopy. Colonies were counted in 5 different fields per well and confirmed by TotalLab Quant v11 software (http://www.totallab.com/).

### Cell invasion assay

The extent of cell invasion was assessed using the BD BioCoat™ Matrigel™ Invasion Chamber (BD Biosciences, Bedford, MA), according to the manufacturer's protocol. A total of 1×10^^5^ cells were seeded onto the inserts (8-µM pore sized polycarbonate membrane) coated with a thin layer of Matrigel Basement Membrane Matrix (BD Biosciences). The inserts were placed into a 24-well plate with 10% serum-containing culture medium or media without serum. The plates were incubated for 48 hrs at 37°C. Cells that invaded the Matrigel matrix to the lower surface of the membrane were fixed and stained with Diff Quik Stain (Dade Behring, Newark, DE, USA) and counted under a light microscope. Four fields in four separate quadrants of each membrane were counted and averaged.

### Statistical Analyses

The continuous data was represented as mean ± standard deviation (S.D.). Two-tailed ANOVA multi-comparison *t*-test was used to assess the difference between mean expression among normal, benign and malignant samples. Pearson correlation test were used for continuous data. A *p*-value <0.05 was considered as significant. The analysis was done by Stat View 5.0 (Cary, NC) and SPSS v 16.0 (Chicago, IL) statistical softwares.

## Results

### KIAA0101 mRNA and protein are highly expressed in adrenocortical neoplasm

**Figure 1:**
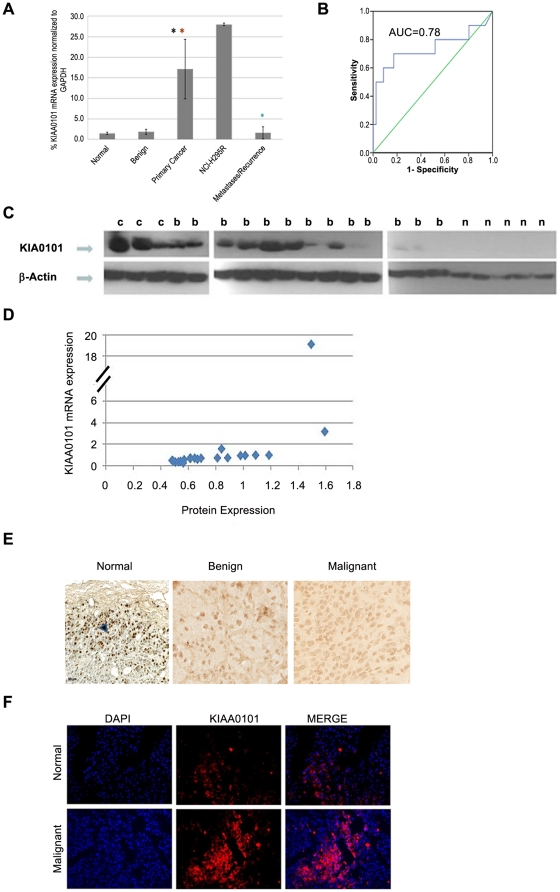
Expression of KIAA0101 mRNA in ACC. **A**) KIAA0101 mRNA expression in normal adrenal cortex (n  =  21), benign adrenocortical tumors (N  =  80), ACC (N  =  10), metastatic and recurrent ACC (n = 30) and the NCI-H295R cell line. KIAA0101 mRNA expression level was determined by quantitative RT-PCR and normalized to GAPDH mRNA expression. Columns represent mean ± standard deviation of replicate determinations. Statistical significant difference is indicated by an asterisk (*) (p<0.05, Kruskal Wallis test). Primary ACC vs. normal (p<0.0001), primary ACC vs. benign adrenocortical tumors (p<0.0001) and primary ACC vs. metastases (p<0.0001). **B**) ROC curve analysis for KIAA0101 mRNA expression. The receiver operating characteristic curve (ROC) is depicted on graph using RT PCR expression data of KIAA0101 normalized to GAPDH in normal adrenal cortex (n  =  21), benign adrenocortical tumors (n  =  80), ACC (n  =  10) . The area under the curve (AUC) was 0.78. A perfect diagnostic marker without any false-negatives or false-positives would have an AUC of 1. **C**) KIAA0101 protein expression in ACC. KIAA0101 protein expression levels in normal adrenal cortex (n = 5), benign (n = 13), and malignant adrenocortical tumors (n = 3) as shown in representative Western blot with KIAA0101 specific and β-Actin control antibodies. β-Actin signal was used as a control for protein loading. C  =  ACC, B  =  benign adrenocortical tumor, and N  =  normal adrenal cortex. **D**) Scatter plot of KIAA0101 mRNA and protein expression level in normal, benign and malignant adrenocortical tissue samples. Y-axis shows normalized mRNA expression by quantitative RT-PCR and X-axis shows protein expression level in the same sample based on band densitometry measurement normalized to β actin expression level. Spearmen correlation coefficient **(r)  = 0.61, p<0.001.** **E**) Immunohistochemistry for KIAA0101 protein expression in normal adrenocortical tissue, benign tumors and ACC. Representative images are shown for each category at 20X magnification. Arrow indicates the positive nuclear staining for KIAA0101. In normal tissue samples, only the subcupsular region was positive. **F**) KIAA0101 cellular localization was analyzed by immunoflouroscence. Nuclei were stained by DAPI (blue), red color indicates KIAA0101 expression. Representative images from malignant ACC and normal samples are shown at 20X magnification.

The expression of KIAA0101 mRNA was significantly higher in ACC as compared to normal adrenocortical tissue and benign adrenocortical tumors (12-fold higher than normal and 9-fold higher than in benign tumors, p<0.0001) (**Figure 1A**). In addition, KIAA0101 mRNA expression level was lower in metastatic and recurrent tumors as compared to primary ACC (p<0.001)

Given the significant expression difference in mRNA between benign and malignant tumors, we were interested in assessing if KIAA0101 was an accurate diagnostic predictor of tumor type. KIAA0101 expression was 84% accurate for distinguishing between ACC and normal and benign adrenocortical tumor samples (number of true positive and negative results divided by the total sample number using a cutoff level of 1.5). The area under the receiver operator characteristic (ROC) curve (AUC) was 0.78 for KIAA0101 mRNA expression (**Figure 1B**), 0.79 for tumor size, and 0.85 for combination of KIAA0101 expression and tumor size.

In addition to high mRNA expression, KIAA0101 protein expression was also elevated in ACC as compared to normal adrenal cortex and benign adrenocortical tumor samples. On Western blot, we found higher expression in ACC (n = 3) than benign adrenocortical tumors (n = 13), and in normal adrenal cortex samples (n = 5) (**Figure 1C**). There was good correlation between KIAA0101 mRNA and protein expression levels (**Figure 1D**, r = 0.61, p<0.001). To further assess KIAA0101 expression, we performed semiquantitative analysis of a tissue array containing small sections of benign and malignant tumors by immunohistochemistry for KIAA0101 protein. This analysis found no significant difference in KIAA0101 protein expression between benign and malignant tumors (p = 0.31). This is not surprising as immunohistochemistry is a semiquantitative method and may not detect smaller differences in protein expression. However, when KIAA0101 protein expression was evaluated in individual benign and malignant samples on the array, significant overexpression was observed as compared to normal samples (**Figure 1E**). KIAA0101 protein was present in the cytoplasm and nucleus, which was also confirmed by immunofluorescence (**Figure 1F**).

**Figure 2:**
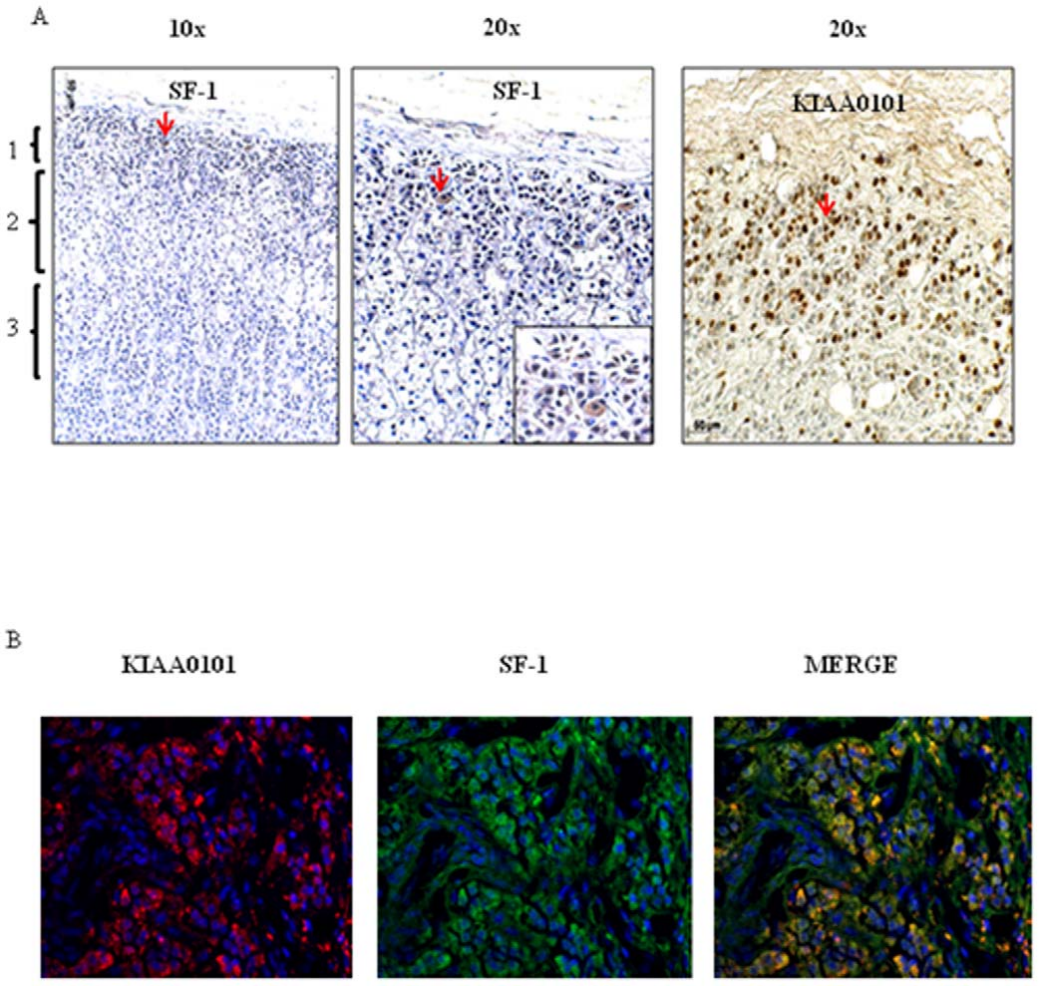
KIAA0101 and Steroidogenic factor 1(SF-1) protein expression in progenitor cells of normal adrenal cortex tissue by immunohistochemistry and immunofluoroscence. **A**) Immunohistochemistry staining of normal adrenal cortex for SF-1 and KIAA0101. In the first panel, the zones of the adrenal cortex are indicated at 10X, 1: Zona glomerulosa containing progenitor cells, 2: Zona fasciculata, 3: Zona Reticularis. Second and third panel indicate the SF-1 (20X with 40X inset) and KIAA0101 positive immunostaining in the Zona Glomerulosa at 20X magnification. Red arrows indicate the positive areas for SF-1 and KIAA0101 staining. **B**) Immunofluoroscence for SF-1 and KIAA0101 in the normal adrenal cortex. Nuclei were stained by DAPI (blue), red color indicates KIAA0101 expression and green color indicates SF-1 expression. Merged Images (yellow color) at 40X show colocalization of the two proteins, SF-1 and KIAA0101 in progenitor cells of normal adrenal cortex.

We observed only sparse KIAA0101 staining in a few normal adrenal cortex samples, which was confined to the subcapsular cortex region (**Figure 2A**). To determine if this focal area positive for KIAA0101 represented adrenal progenitor cells, we co-stained the same normal adrenal cortex samples with steroidogenic factor 1 (SF-1) antibody, a marker of adrenal progenitor cells [23]. Interestingly, normal adrenal cortex samples showed co-staining of KIAA0101 and SF-1 together in the subcapsular or progenitor region of the adrenal cortex (**Figure 2B**).

### Mechanism of KIAA0101 overexpression in ACC

To determine whether the KIAA0101 overexpression was a consequence of gene sequence variations, we performed mutation analysis in the KIAA0101. We found one de *novo* G>A transition mutation at position 186 which falls in non-coding region of KIAA0101 in a benign sample. We also observed one polymorphism in the 5′ UTR in exon 1–2 (88T>C). About 6.6% of benign were heterozygous and 12.5% in the normal but none in malignant samples (**Table 2**). Given these results it is unlikely that the KIAA0101 overexpression is a result of mutation.

**Table 2:** Summary of sequencing changes in KIAA0101.

Variations	Position	Region	Distribution		
			Normal (n = 8)*	Benign (n = 75)*	Malignant (n = 10)
88T>C	EXON 1–2	5′ UTR	1/8 (12.5%)	5/75 (6.6%)	ND
410T>C	EXON 1–2	Intronic	1/8 (12.5%)	ND**	ND
15360T>C	EXON 4	Intronic	ND**	1/75 (1.3%)	ND

*Percentage of mutation is for the samples which were analyzed by category and available for sequencing.

**ND (not detected).

### The functional effect of knockdown of KIAA0101 gene in an ACC cell line

**Figure 3:**
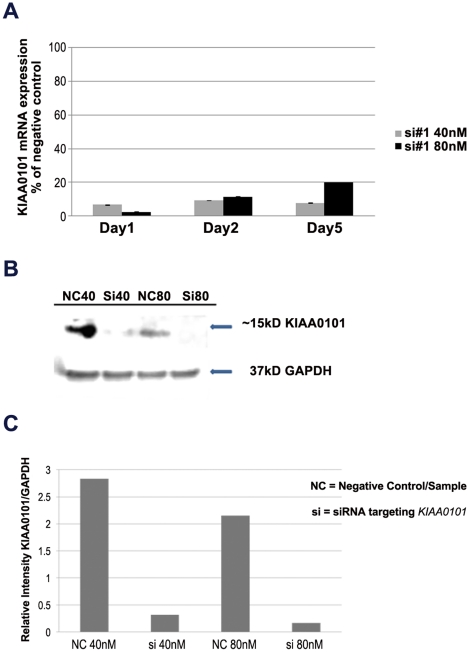
KIAA0101 mRNA and protein expressions were decreased with siRNA knockdown in the NCI-H295R adrenocortical carcinoma cell line. **A**) H295R cells were transfected with KIAA0101 siRNA and negative control and analyzed for KIAA0101 mRNA expression after 48 hours of treatment. Columns represent KIAA0101 mRNA expression percentage relative to negative control ± standard deviation of four experiments. **B**) Representative Western blot demonstrating protein expression of KIAA0101 after 6 days of siRNA and negative control. **C**) Relative intensity of western blot using Image J Software. Columns indicate ratio of KIAA0101 and GAPDH intensity measurements. NC40 = Negative control at 40 nM; SI40  =  siRNA at 40 nM; NC80  =  Negative control at 80 nM; SI80  =  siRNA at 80 nM.

**Figure 4:**
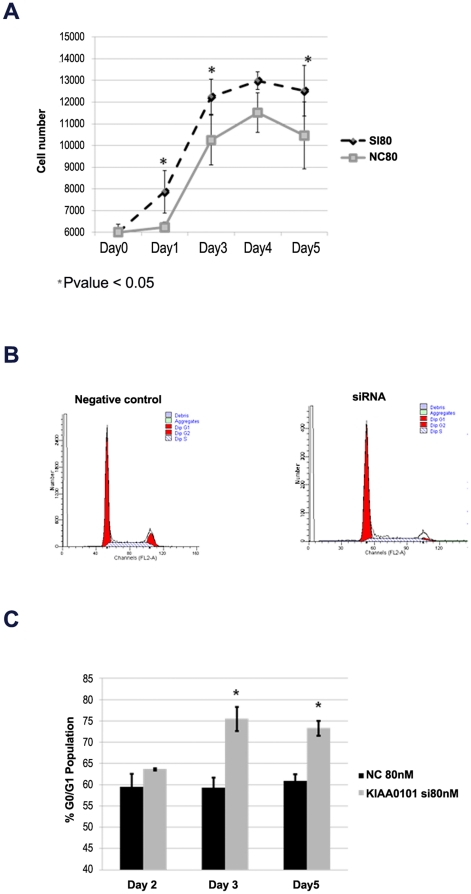
Cell proliferation increased with KIAA0101 siRNA knockdown in the NCI-H295R cell line. **A**) The number of cells for the KIAA0101 siRNA treated and negative control treated groups are shown at indicated time points (significant values are indicated by asterisk (*) (p  =  0.005; relative to negative control). Error bars represent ± standard error of mean and is representative of four experiments. NC80 = Negative control at 80 nM; SI80  = siRNA at 80 nM. **B**) KIAA0101 gene knockdown and cell cycle analysis is indicated at different time points. Representative image of cell cycle profile from siRNA and negative control treatment groups. **C**) Each column indicates the distribution of G0/G1 population of cells in each group at various time points. Error bars represent ± standard error of mean and is representative of four experiments.*Day 3 and 5, p<0.05; relative to negative control.

Given the significant overexpression of KIAA0101 in ACC (**Figure 1**) and the observation that KIAA0101 expression is restricted to proliferating adrenal progentitor cells (**Figure 2B**), we hypothesized that KIAA0101 plays a role in adrenocortical cell growth. To address this, we used siRNA to knockdown KIAA0101 expression in the ACC cell line, NCI-H295R. Transient transfection of KIAA0101 specific siRNA resulted in a 10-fold knockdown within 24 hours and this effect was sustained for up to 6 days at the mRNA and 80% reduced at the protein levels as compared to a non targeting siRNA (negative control siRNA) (**Figure 3**). KIAA0101 knockdown resulted in modest increase in cellular proliferation with an increase in cell number of up to 25% (p = 0.005) (**Figure 4A**). Also, the cell line doubling time and growth rate were different for siRNA knockdown as compared to the negative control (average doubling time of 4.7 and growth rate of 0.14 as compared to average doubling time of 3.5 and growth rate of 0.19 from day 1 to 5, respectively). Cell cycle analysis revealed a modest increase in the number of cells in G1 phase (p = 0.013) (**Figure 4B**). Considering these contradictory results, we also evaluated mRNA expression of G1 cell cycle regulatory proteins at G1 phase after KIAA0101 knockdown. KIAA0101 knockdown did not significantly alter the mRNA expression of p21 and Cyclin D1, cell cycle regulatory proteins, in an ACC cell line. In addition, KIAA0101 gene knockdown had no significant effect on the number of cells in apoptosis (data not shown).

**Figure 5:**
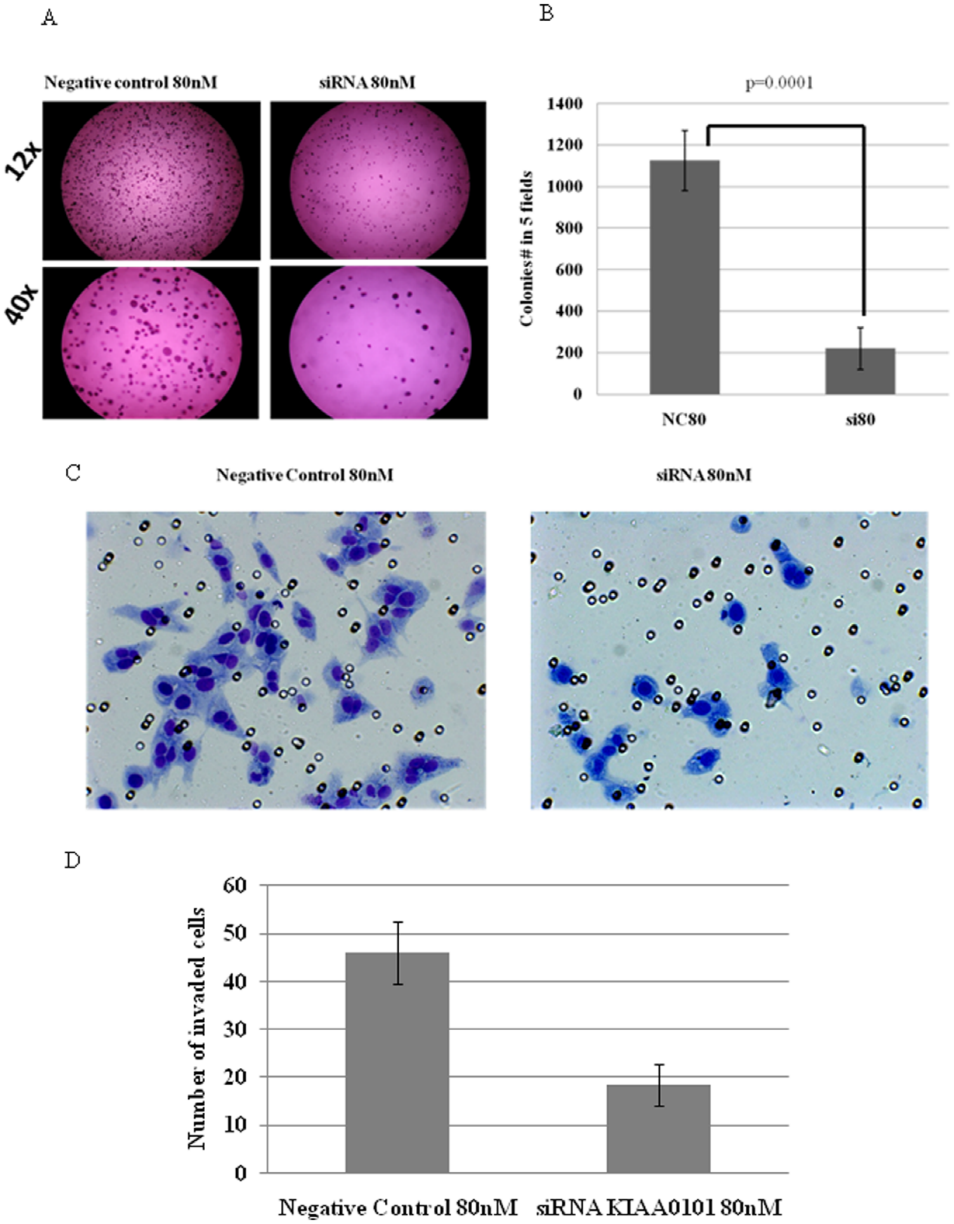
KIAA0101 siRNA knockdown reduced soft agar anchorage independent growth and invasion in NCI-H295R cells. **A**) Cells cultured for 15 days in the presence of indicated concentrations of siRNA and negative control. Representative images are shown at 12X and 40X magnifications. **B**) The distribution and number of colonies in group treated with KIAA0101 siRNA and negative control group at 80 nM (p = 0.001; relative to negative control; NC80 =  Negative control at 80 nM; SI80  = siRNA at 80 nM). Error bars represent ± standard error of mean and is representative of four experiments. **C**) Invading cells were stained with 0.2% crystal violet and visualized by microscopy. Representative images at 20X following invasion assay. **D**) The distribution of number of cells in KIAA0101 siRNA and negative control group. Columns represent the mean ± standard deviation (SD) of three independent experiments performed in triplicate. *p<0.05 KIAA0101 siRNA vs. negative control. H295R cells were transfected with negative control or KIAA0101 siRNA at 80 nM for 120 h, followed by an invasion assay for 48 h.

Given the paradoxical effects, a modest increase in cellular proliferation and G1 arrest, we used additional functional assays to better understand the phenotypic changes associated with KIAA0101 knockdown. Specifically tumorogenicity and metastatic potential were evaluated. KIAA0101 knockdown in NCI-H295R cells reduced the number of colonies that were able to grow in soft agar by almost 80% (p = 0.001) at 16 days of culture (**Figures 5A and 5B**) with a 60% reduction in cell invasion in the NCI-H295R cell line (p = 0.006) (**Figures 5C and 5D**).

## Discussion

In this study, we examined KIAA0101 expression and function in ACC. Our results indicate that KIAA0101 is overexpressed in ACC, is a good diagnostic marker for ACC, and is a marker of cellular proliferation. Suppressing KIAA0101 expression in adrenocortical carcinoma cells resulted in suppression of growth and invasion, suggesting that it plays a growth promoting role in ACC.

Previous studies have demonstrated altered expression of KIAA0101 in human malignancies and absent or low expression in normal tissue [12], [13], [14], [15], [16], [17], [18], [19], [20], [21], [22]. However, conflicting results have been obtained for KIAA0101 expression in hepatocellular carcinoma (HCC). In one study, KIAA0101 expression was found to be downregulated in HCC [13]. Conversely, in a separate study, in a large cohort of patients with HCC, KIAA0101 was upregulated[18]. Our study also demonstrated the KIAA0101 overexpression in primary ACC but, interestingly, KIAA0101 mRNA expression was lower in tumors from a cohort of patients with metastatic and recurrent ACCs who responded to systemic chemotherapy and underwent resection. These results suggest that KIAA0101 expression may vary as a result of treatment and could possibly be used as a biomarker for treatment response in ACC and other cancers.

Although we found that KIAA0101 protein expression was higher in malignant and benign tumors as compared to normal samples, we did detect KIAA0101 expression in a subset of normal samples. Its expression was confined to the subcapular region of the adrenal cortex and overlapped the SF-1 positive staining cells. In this region, SF-1 positive cells are hypothesized to be adrenal progenitor cells [24]. Progenitor cells possess high proliferative capacity. KIAA0101 and SF-1 co-expression in the progenitor region of normal adrenal cortex suggests that KIAA0101 may be a marker of proliferation in adrenocortical cells. Consistent with this, a study by Simpson et al., has demonstrated the localization of KIAA0101 in basal cell layer of the skin and the proliferative zone in the crypts of the colon[15].

Little is known about the function of the KIAA0101 gene in tumor cell biology. Previously, studies have suggested that KIAA0101 is involved in the regulation of DNA replication, cell cycle, apoptosis and cellular proliferation [13], [14], [15], [18], [25], [26]. Given that KIAA0101, under basal culture conditions, was highly expressed in the NCI-H295R cell line, we used a gene silencing strategy to effectively knockdown its expression. Using this strategy, we observed a modest increase in cellular proliferation, suggesting that KIAA0101 may have a growth suppressive function in ACC. In addition to the growth regulatory function of the *KIAA0101* gene, we observed that KIAA0101 knockdown slightly increased the number of cells in G1 phase with a concomitant decrease in the number of cells in S phase. This result suggests that KIAA0101 may function as a cell cycle checkpoint protein for G1/S phase cell cycle transition. Indeed, KIAA0101 encodes a 15-kDa protein, which contains a conserved proliferating cell nuclear antigen (PCNA) binding motif, and was initially discovered in a study screening for binding partners for the PCNA in yeast [20]. KIAA0101 shares the PCNA binding motif with many important cell cycle regulatory PCNA binding proteins such as p21, p57 and p33ING1b [27]. KIAA0101 also competes with p21 for binding to PCNA and regulate cell cycle progression [20]. In our study, it is possible that after knockdown of KIAA0101, p21 may bind to PCNA and leads to G1/S arrest. However, given the modest effect on cell cycle progression, we did not find significant influence on p21 and CyclinD1 levels of G1 cell cycle phase proteins. On the other hand, knockdown of KIAA0101 greatly reduced anchorage independent growth and invasion. It strongly indicates that KIAA0101 expression in adrenocortical tumor cells might contribute to tumor progression. These seemingly contradictory effects are consistent with previous observations [13], [14], [18]. A study by Hosokowa et al. demonstrated the potential oncogenic role of KIAA0101 in pancreatic cancer [14]. They overexpressed KIAA0101 exogenously in NIH3T3 fibroblast cells which revealed in vivo cancer cell growth, confirming its growth-promoting and oncogenic nature. Similarly, in HCC, it has been shown that KIAA0101 overexpression was associated with higher vascular invasion and contributes to poor prognosis [18]. However, in another study of HCC, KIAA0101 was downregulated and found to be a growth-inhibitory gene [13]. Specifically, depending on the tumor type and cell line studied, KIAA0101 has been found to have both growth inhibitory and stimulatory effects [13], [14], [15], [16], [18], [20]. Such paradoxical effects on growth and cell cycle progression (growth promoting or inhibiting effects) of key cell cycle regulating genes, such as p21 and p27, have also been observed depending on cell type and external cell stimuli or stress factors [28].

While most genes upregulated in malignancy have a growth promoting function, one of the most common tumor suppressor genes in human malignancy, p53, is overexpressed and has a tumor suppressor function because of inactivating dominant negative mutations. In our study, KIAA0101 was also overexpressed, therefore, we hypothesized that sequence alterations may reveal the reason for dysregulation. However, we did not detect the presence of such mutations in coding region of KIAA0101 consistent with one other study [20]. However, previous studies have revealed that the genomic region of KIAA0101, chromosomal locus 15q is frequently gained in ACC [29], [30]. Additional studies in a large set of ACCs will be necessary to determine the molecular mechanism for KIAA0101 overexpression.

In summary, the results of our study provide the first evidence that KIAA0101 is a marker of cell proliferation and is overexpressed in ACC, therefore it suggests that its expression could be used as a molecular marker for the diagnosis of ACC. In addition, our functional studies suggests, it has modest effect on the cell proliferation and cell cycle progression. However, the majority of the results support that it is a potential tumor-promoting gene and contributes to metastatic potential in vitro.
